# Soluble P-Selectin and von Willebrand Factor Rise in Healthy Volunteers Following Non-exertional Ascent to High Altitude

**DOI:** 10.3389/fphys.2022.825819

**Published:** 2022-02-16

**Authors:** Helena A. Turton, Josephine Pickworth, Gordon G. Paterson, Allan Lawrie, J. Kenneth Baillie, A. A. Roger Thompson

**Affiliations:** ^1^Department of Infection, Immunity and Cardiovascular Disease, University of Sheffield, Sheffield, United Kingdom; ^2^Apex (Altitude Physiology Expeditions, SC030345), Edinburgh, United Kingdom; ^3^Centre for Primary Care and Public Health, Blizard Institute, Barts and the London School of Medicine and Dentistry, Queen Mary University of London, London, United Kingdom; ^4^Department of Anaesthesia, Critical Care and Pain Medicine, University of Edinburgh, Edinburgh, United Kingdom; ^5^Sheffield Teaching Hospitals NHS Foundation Trust, Sheffield, United Kingdom

**Keywords:** altitude, hypoxia, P-selectin, von Willebrand factor, platelet activation, coagulation, thrombosis

## Abstract

Reduced oxygen tensions experienced at high altitudes are thought to predispose to thrombosis, yet there are few studies linking hypoxia, platelet activation, and thrombosis. Reports of platelet phenotypes in hypoxia are inconsistent, perhaps due to differing degrees of hypoxia experienced and the duration of exposure. This study aimed to investigate the relationship between soluble P-selectin, a marker of platelet activation, and von Willebrand factor (vWF) on exposure to hypoxia. We measured plasma concentrations of P-selectin and vWF in sixteen healthy volunteers before, during and after the APEX 2 expedition. APEX 2 consisted of a non-exertional ascent to 5,200 m, followed by 7 consecutive days at high altitude. We showed that high altitude significantly increased mean plasma P-selectin and vWF compared to pre-expedition levels. Both plasma marker levels returned to baseline post-expedition. We found a strong positive correlation between vWF and P-selectin, but no association between P-selectin and platelet count. Our results are consistent with previous work showing evidence of platelet activation at high altitude and demonstrate that the rise in P-selectin is not simply due to an increase in platelet count. As vWF and P-selectin could be derived from either platelets or endothelial cells, further work assessing more specific markers of endothelial activation is proposed to provide insight into the source of these potential pro-thrombotic biomarkers at altitude.

## Introduction

Stroke or venous thrombosis have been reported on exposure to high altitude. Indeed, the risk of these thrombotic events was reported to be 30-fold higher during prolonged exposure to altitudes greater than 3,000 m (Anand et al., 2001). Platelets, together with endothelial cells (ECs), and numerous circulating coagulation factors, are the fundamental mediators of coagulation and thrombosis. The success of antiplatelet therapy in patients with increased thrombotic risk provides strong evidence for the importance of these cells in thrombosis. During platelet activation, platelet α-granules release the contents of hundreds of bioactive molecules (i.e., ADP, serotonin, and thromboxane) *via* exocytosis (van der Meijden and Heemskerk, 2019). To further potentiate platelet activation, adhesive receptors that were once located on intracellular α-granule membranes at rest are expressed on the platelet plasma membranes. Following platelet activation, proteolytic cleaving/shedding regulates the surface expression of around 10% of all platelet receptors, of which P-selectin is one of the most abundantly expressed in the platelet “sheddome” (Fong et al., 2011). Specifically, interaction of P-selectin with endothelial PSGL-1 and alternative splicing of P-selectin liberate P-selectin and in turn, soluble P-selectin (sP-selectin) has been exploited as a biomarker of platelet activation in normoxic settings (Ishiwata et al., 1994; Dole et al., 2007).

Platelet activation leading to platelet aggregation and blood coagulation is a tightly regulated process. Blood coagulation stops bleeding, but subsequently, clot propagation must be regulated and resolved to prevent occlusion of vessels. ECs control many pathophysiological responses including coagulation. For instance, ECs can produce antithrombotic, pro-fibrinolytic factors, and metalloproteases to cleave platelet aggregates. Hypoxia-induced reactive oxygen species destroy EC microtubules *via* PI3K/stathmin1 pathway (Cao et al., 2019). Thus, malfunctioning ECs may contribute to hypercoagulation, thrombosis, and cardiovascular diseases (Poredos and Jezovnik, 2018). Hypoxia also induces exocytosis of Weibel-Palade bodies (WPb) from ECs, which contain von Willebrand Factor (vWF) (Pinsky et al., 1996) and clinical studies suggest that increased plasma vWF is a biomarker of endothelial dysfunction in cardiovascular disease (Conway et al., 2002; Horvath et al., 2004).

Recent reports of hypoxic alterations in the platelet proteome and platelet purinergic signaling, and increased platelet aggregation to a fixed dose of ADP indicate that hypoxia alters platelet function, and this could increase thrombosis risk at high altitude (Tyagi et al., 2014; Rocke et al., 2018; Paterson et al., 2020; Shang et al., 2020). In addition, biomarker approaches have quantified markers of increased platelet activation upon high altitude exposure (Lehmann et al., 2006; Lackermair et al., 2019). In previous work we showed a rise in platelet count during a 7-day sojourn at 5,200 m (Paterson et al., 2020). Therefore, we sought to explore the relationship between markers of platelet activation (sP-selectin), endothelial dysfunction (vWF) and platelet count.

## Methods

**FIGURE 1:**
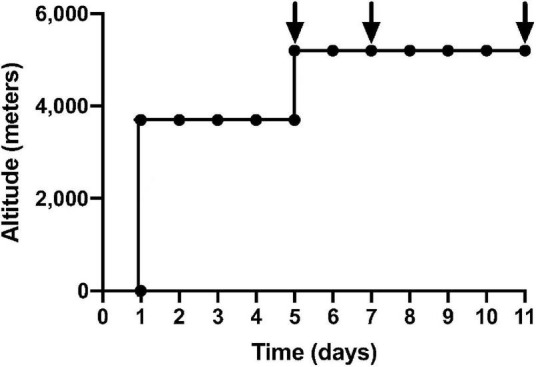
Ascent profile of APEX 2 expedition: black arrows indicate sampling on day 1, 3, 7 of 7 consecutive days at an altitude of 5,200 m. The first 5 days were spent in La Paz, Bolivia (3,600 m), before a non-executional ascent to Chacaltaya Laboratory (5,200 m) over 90 min *via* road.

Stored samples from 16 healthy participants from the APEX 2 expedition were used in this experiment. All participants were resident at altitudes less than 600 m and had not been to heights greater than 1,500 m in the 3 months preceding the study. All participants had no known cardiovascular or respiratory conditions and no pre-existing coagulopathy. Participants were asked to refrain from alcohol for the duration of the study. These participants were in the placebo arm of previously published randomized trials (Baillie et al., 2009; Bates et al., 2011). The study was approved by the Lothian Research Ethics Committee and all participants gave informed consent in keeping with the Declaration of Helsinki. Participants flew to La Paz, Bolivia (3,600 m) and spent 4 or 5 days there before a 90-min non-exertional ascent, *via* road, to the Chacaltaya Laboratory (5,200 m). From day 5 of the expedition, 7 consecutive days were spent at 5,200 m, where full blood count and blood samples were taken on days 1, 3, and 7 (Figure 1). Sea-level samples were taken pre-expedition and 11–16 weeks post-expedition. Venepuncture was performed using 21G needles into sterile citrated tubes. Samples were immediately centrifuged for 10 min and plasma aliquots were transported on dry ice by specialist international courier and stored at −80°C prior to analysis. P-selectin and vWF in a fixed volume (50 μL) of citrated plasma were quantified using Luminex xMAP technology (Luminex 200) in coordination with R&D Systems’ Human Premixed Multi-Analyte Kit Luminex immunoassay. Median fluorescence intensities were exported from Luminex xPONENT 4.3 software and were fitted to a six-point standard curve generated using four-parameter logistic regression, prior to applying dilution factors. ROUT outlier analysis identified two outliers in the data set, which were removed. Mixed-effects model ANOVAs and *post-hoc* Dunnett’s multiple comparisons testing were applied to compare mean vWF and sP-selectin. A linear regression model assessed the correlation of sP-selectin and vWF and P-selectin and platelet count. Adjusted *p*-values, using Bonferroni corrections, were reported.

## Results

The mean age of participants was 21 ± 2. Body mass index was within a narrow range at 23.2 kg/m^2^ (±2) and participants had normal lung function during baseline tests [forced expired volume in 1 s, 4.03 L (±1) and forced vital capacity, 4.86 L (±1)]. Physiological and hematological parameters at sea level and altitude are provided in Table 1.

**TABLE 1:** Physiological and hematological parameters of study participants at sea-level and altitude.

	Sea level pre-expedition	Day 1 at 5,200 m	Day 3 at 5,200 m	Day 7 at 5,200 m	Post-expedition
Heart rate (bpm)	71.8 (±9.6)	98.3 (±18.8)*	112.3 (±19.1)*	104.2 (±14.5)*	78 (±17.5)
SaO_2_ (%)	98.3 (±1.4)	78.3 (±5.1)*	74.1 (±5.1)*	76.9 (±6.0)*	98.8 (±1.2)
Hemoglobin (g/dL)	14.3 (±1.1)	15.9 (±1.9)*	16.4 (±1.6)*	16.2 (±1.6)*	13.8 (±0.9)
Hematocrit (%)	44.0 (±2.4)	47.7 (±6.1)*	49.7 (±5.1)*	49.1 (±4.9)*	40.2 (±2.6)*
Platelets (×10^9^/ml)	249.2 (±55.6)	238.7 (±57)	252.6 (±52.6)	288.2 (±65.5)*	248.2 (±51)
sP-selectin (pg/ml)	15,736 (±4,811)	19,454 (±4,116)*	20,176 (±4,470)*	21,508 (±5,469)*	18,738 (±5,146) *n* = 15
vWF (pg/ml)	278.7 (±75.8) *n* = 15	357.8 (±61.2)*	367 (±69.3)*	368 (±76.2)*	325.9 (±75.4)

*Data are mean ± SD.*

*SaO_2_, peripheral oxygen saturation; vWF, von Willebrand Factor.*

*p < 0.05 vs. sea level pre-expedition values by mixed-effects ANOVA and Dunnett’s multiple comparisons test. n = 16 unless stated.

**FIGURE 2:**
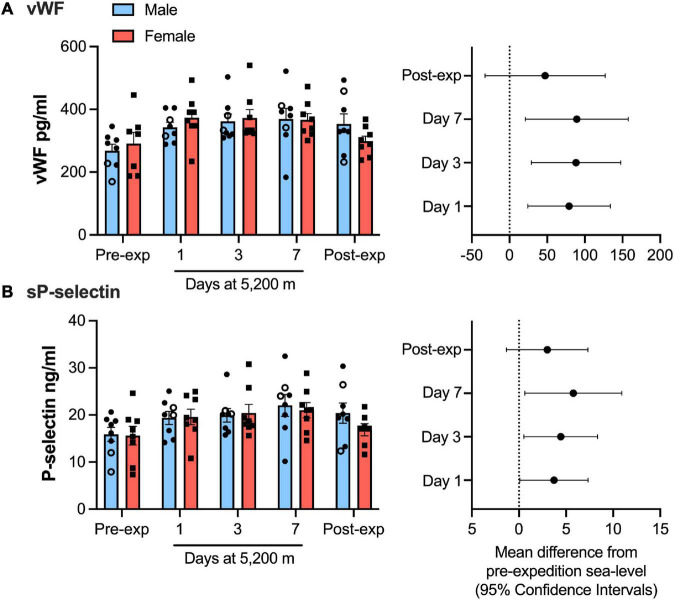
von Willebrand factor (vWF) and P-selection plasma levels significantly increased during high-altitude induced hypoxia. Luminex immunoassay measurements demonstrate Plasma vWF **(A)** and soluble P-selection **(B)** levels at sea level and altitude split by sex. Left hand panels show mean data (top of bars) ± SEM with each individual value represented dots. Two smokers are indicated by open circles. Right hand pannels indicate mean difference from pre-expedition sea level with Confidence Intervals calculated by mixed model ANOVA and Dunnett’s multiple comparisons test *n* = 16.

**FIGURE 3:**
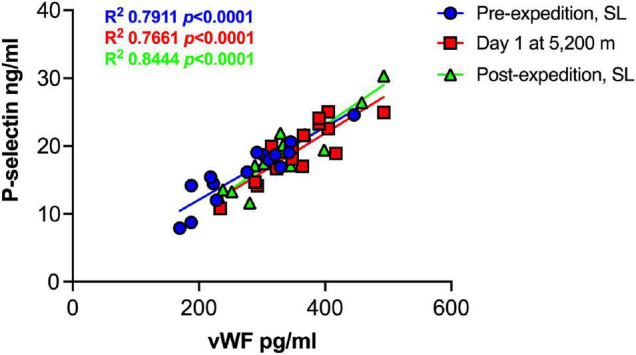
Strong positive correlation between Plasma P-selection and von Willebrand Factor (vWF) at sea level and during high altitude induced hypoxia. Plasma vWF and P-selectin assessed by Luminex immunoassay (*n* = 16). SL; sea level.

**FIGURE 4:**
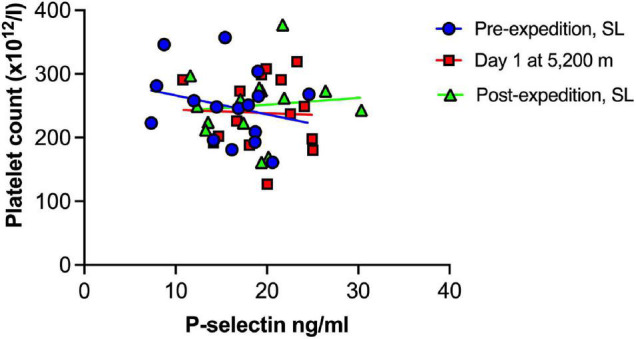
No association between Plasma P-selectin and platelet count at sea level or during high altitude induced hypoxia. Plasma P-selection assessed by Luminex immunoassay (*n* = 16). SL, sea level.

Plasma vWF significantly increased upon altitude exposure (Figure 2 and Table 1) compared to pre-expedition sea-level values. sP-selectin was also significantly higher by day 1 at 5,200 m and levels of both sP-selectin and vWF remained elevated during the altitude sojourn compared to pre-expedition sea-level values (Figure 2). Plasma vWF and sP-selectin were not significantly different from baseline pre-expedition levels on return to sea level, 11–16 weeks post expedition, nor was there a difference between males and females at any time point. We found no correlation between sP-selectin or vWF and body mass index (data not shown). A strong significant correlation was observed between vWF and P-selectin in plasma, irrespective of time at altitude (Figure 3). P-selectin and platelet count were not correlated at sea-level or upon altitude-induced hypoxia (Figure 4).

## Discussion

This study reports increases in sP-selectin and vWF following ascent to 5,200 m and these changes did not correlate with platelet count. The findings are in keeping with reports of increased platelet activation at altitude (Rocke et al., 2018; Paterson et al., 2020) and mirror previous findings of increased plasma sP-selectin at high altitude (Lehmann et al., 2006; Lackermair et al., 2019). Our study eliminates potential confounding factors such as exertional ascent and sampling method. For example, Lackermair et al. (2019) measured sP-selectin in serum rather than plasma; an experimental design reported to artifactually increase sP-selectin level (Caine and Blann, 2003). Lackermair et al. (2019) reported increased sP-selectin after a substantially shorter exposure time in hypoxia (30 min) compared to our findings, which showed plasma sP-selectin to increase within 24 h of ascent to 5,200 m. Our findings showed that levels of sP-selectin remained high during a 1-week high-altitude sojourn. In contrast to the study by Lehmann et al. (2006), in which participants had increased plasma sP-selectin after a combined climb of 5.5 h over 2 days to reach altitude, we found evidence of platelet activation despite a non-exertional ascent, supporting the hypothesis that hypoxia rather than exertion promoted the increase in sP-selectin (Ahmadizad and El-Sayed, 2003).

A rise in platelet count at altitude has been previously reported by our group and others (Hudson et al., 1999; Rocke et al., 2018; Paterson et al., 2020), however increased sP-selectin was not associated with platelet count in the current study. This supports the emerging view that platelet activation is not merely due to increased platelet number in hypoxia, but rather the contents, function and metabolism of hypoxic platelets differs from platelets in a normoxic microenvironment (Tyagi et al., 2014; Cameron et al., 2018; Shang et al., 2020).

We found an increase in vWF that correlated strongly with sP-selectin levels. Weibel-Palade bodies are exported from hypoxic endothelial cells and may contain both vWF and P-selectin (Pinsky et al., 1996). Both biomarkers are also contained and released by platelets, indeed in healthy individuals, ∼20% of plasma vWF is derived from platelets (Sporn et al., 1985). The source of vWF may impact upon its function, for example endothelial-derived vWF was the dominant contributor to thrombus growth and infarct size in a brain ischemic/reperfusion model, based on experiments using bone marrow transplant chimeras of vWF knockout and WT mice (Dhanesha et al., 2016). However, the methods used in our study cannot define the source of the biomarkers. Future work could test more specific markers or assess isolated platelets. Interestingly, a recent study using electron microscopy on isolated platelets from subjects who had lived at altitude (above 3,600 m) for more than 3 months, demonstrated fewer alpha granules in platelets from hypoxic subjects than under normoxic conditions, suggesting greater degranulation at altitude (Shang et al., 2020). This, alongside upregulated P-selectin protein levels and increased levels of other alpha granule proteins in plasma (platelet factor 4) from these subjects, suggest that the changes we observed may be followed by persisting alterations in the platelet proteome (Shang et al., 2020).

Our study has several limitations. Changes in time zone, diet and sleep disturbance could act as potential confounders that impact upon platelet activation or endothelial function (Von Känel et al., 2007; Scheer et al., 2011; Zhu et al., 2021). Furthermore, as noted above, additional biomarkers and assays on fresh isolated platelets could provide further insight into the source of elevated sP-selectin and vWF levels. Finally, due to methodological differences that limit comparability of our measurements to previous studies, it is unclear whether the rise observed in vWF and sP-selectin in our study would mirror those observed in the context of other pathologies. For example, the rise in vWF is likely to be greater following acute myocardial infarction than the rise observed in our study (Sakai et al., 2000), and it is unlikely that the magnitude of change in sP-selectin is equivalent to the 4-fold rise observed in disseminated intravascular coagulation (Chong et al., 1994).

Nonetheless, higher levels of vWF and sP-selectin at altitude are consistent with the hypothesis that altitude exposure may increase risk of thrombosis. Increases in plasma vWF induced by a single nucleotide polymorphism (rs1063856) in exon eight of vWF are associated with a higher risk of venous thrombosis (Smith et al., 2011). Furthermore, vWF levels were associated with cardiovascular events, stroke, mortality in anticoagulated patients with atrial fibrillation (García-Fernández et al., 2017). The link between sP-selectin levels and risk of future cardiovascular events is less clear (Conway et al., 2002) but levels of sP-selectin have been reported to predict risks of future venous thromboembolic events (Kyrle et al., 2007).

To conclude, our study provides further evidence of hypoxia-induced alterations in coagulation, but further studies are required to clarify the source of increased vWF and sP-selectin and to test the utility of these biomarkers in the prediction of thrombotic risk at altitude.

## Data Availability Statement

The raw data supporting the conclusions of this article will be made available by the authors, without undue reservation.

## Ethics Statement

The studies involving human participants were reviewed and approved by the Lothian Research Ethics Committee. The patients/participants provided their written informed consent to participate in this study.

## Author Contributions

AT, GP, and JB: conception of the study. HT, JP, GP, and AT: data analysis. HT, JP, GP, AL, JB, and AT: data interpretation. HT and AT: writing. All authors critically reviewed and revised the manuscript.

## Conflict of Interest

The authors declare that the research was conducted in the absence of any commercial or financial relationships that could be construed as a potential conflict of interest.

## Publisher’s Note

All claims expressed in this article are solely those of the authors and do not necessarily represent those of their affiliated organizations, or those of the publisher, the editors and the reviewers. Any product that may be evaluated in this article, or claim that may be made by its manufacturer, is not guaranteed or endorsed by the publisher.
